# A qualitative study to understand how Ebola Virus Disease affected nutrition in Sierra Leone—A food value-chain framework for improving future response strategies

**DOI:** 10.1371/journal.pntd.0007645

**Published:** 2019-09-10

**Authors:** Stephen R. Kodish, Frank Bio, Rachel Oemcke, James Conteh, Jean Max Beauliere, Solade Pyne-Bailey, Fabian Rohner, Ismael Ngnie-Teta, Mohammad B. Jalloh, James P. Wirth

**Affiliations:** 1 GroundWork, Hintergasse, Fläsch, Switzerland; 2 FOCUS 1000, Freetown, Sierra Leone; 3 United Nations Children’s Fund (UNICEF), Conakry, Guinea; 4 Sierra Leone Ministry of Health, Freetown, Sierra Leone; Medizinische Universitat Wien, AUSTRIA

## Abstract

**Background:**

This study sought understand how the 2014–2016 EVD Virus Disease (EVD) outbreak impacted the nutrition sector in Sierra Leone and use findings for improving nutrition responses during future outbreaks of this magnitude.

**Methodology:**

This qualitative study was iterative and emergent. In-depth interviews (*n* = 42) were conducted over two phases by purposively sampling both *key informants* (*n* = 21; government stakeholders, management staff from United Nations (UN) agencies and non-governmental organizations (NGO)), as well as *community informants* (*n* = 21; EVD survivors, health workers, community leaders) until data saturation. Multiple analysts collaborated in a team-based coding approach to identify key themes using Dedoose software. Findings are presented as both quotations and tables/figures.

**Results:**

The EVD outbreak effects and the related response strategies, especially movement restriction policies including 21-day quarantines, contributed to disruptions across the food value-chain in Sierra Leone. System-wide impacts were similar to those typically seen in large-scale disasters such as earthquakes. Participants described an array of direct and indirect effects on agricultural production and food storage and processing, as well as on distribution, transport, trade, and retailing. Secondary data were triangulated by interviews which described the aggregate negative effect of this outbreak on key pillars of food security, infant and young child feeding practices, and nutrition. During the humanitarian response, nutrition-specific interventions, including food assistance, were highly accepted, although sharing was reported. Despite EVD impacts across the entire food value-chain, nutrition-sensitive interventions were not central to the initial response as EVD containment and survival took priority. Culturally-appropriate social and behavior change communications were a critical response component for improving health, nutrition, and hygiene-related behaviors through community engagement.

**Conclusions:**

Infectious diseases such as EVD have far-reaching effects that impact health and nutrition through interrelated pathways. In Sierra Leone, the entire food value-chain was broken to the extent that the system-wide damage was on par with that typically resulting from large natural disasters. A food value-chain approach, at minimum, offers a foundational framework from which to position nutrition preparedness and response efforts for outbreaks in similar resource constrained settings.

## Author summary

The 2014–2016 EVD outbreak has greatly impacted the population health and nutrition of affected countries in West Africa, including that of Sierra Leone. Since this recent outbreak, the humanitarian community acknowledges the need for improved solutions to better prepare for, and respond. Despite the importance of nutrition during outbreaks, there has been little systematic research conducted for understanding lessons learned and improving upon the typical nutrition response options currently available. This study used qualitative interviews to collect in-depth narratives from government officials, front-line health workers, non-government organization management, and community members including local leaders and EVD survivors. Findings reveal the unprecedented magnitude of this outbreak, which had systems-wide impacts not dissimilar to those felt by natural disasters. Interviews with people who lived through this event in Sierra Leone described EVD effects which revealed the importance and fragility of multiple, interconnected systems comprising the food value-chain for optimal nutrition in Sierra Leone. Findings across the food value-chain reveal how this interconnected system was impacted at every level with consequences for population-level nutrition. In preparation for future outbreaks of this magnitude, such a framework may prove useful for policy and planning, including improved guidelines development for employing coordinated nutrition-specific *and* nutrition–sensitive approaches that address immediate and underlying determinants of nutritional status.

## Introduction

The first three human outbreaks of Ebola Virus Disease (EVD), a zoonotic disease, were recorded between 1976–1979 in the Democratic Republic of Congo and Sudan, with fewer than 400 deaths total. Since the first human case in 1976 until 2013, just thirteen human outbreaks from various EVD subtypes (*E*. *Sudan*, *E*. *Zaire*, *E*. *Ivory Coast*) had occurred in Africa, directly causing an estimated 1,300 deaths [1]. Understanding this natural history of EVD underscores the uniquely severe and widespread nature of the 2014–2016 outbreak, whose reported number of deaths surpassed the cumulative sum of EVD outbreaks from the previous 32 years (1976–2008) [2].

Since December 2013, when the first case was identified in rural Guinea, until today–years after the final reported case–the 2014–2016 EVD outbreak has had a lasting impact on the population health, livelihoods, and social dynamics of affected countries in West Africa [3]. In Sierra Leone alone, an estimated 14,124 cases and 3,956 deaths were a direct consequence of the outbreak [4]. And today, thousands of survivors are still coping with the prolonged effects of their own illness episodes [5]. A large proportion of this disease burden may be ascribed to well-documented structural and social challenges that inhibited a timely and effective response at large [6].

Much of it may also be attributable to an infectious disease outbreak in an economically developing country, yet still under resourced with fragile health and nutrition situations. Prior to the outbreak, Sierra Leone was effectively emerging from civil war and unrest, seeing 13.5% household income gains since 2001 [2]. There was increasingly strong trade of food commodities: livestock with Guinea and re-imported rice, as well as seasonal livestock with Liberia, for example [7]. From 2008 to 2013, the proportion of underweight children had decreased from 21 to 16 percent, a key indicator of an improving nutrition situation [8]. Despite such gains, the population majority was living at or below the absolute poverty line and reliant on agricultural livelihoods, even in Freetown where urban and peri-urban agriculture was practiced [2, 9]. In 2013, 38% and 9% of children under five years of age remained stunted and wasted, respectively; and only 32% of children under 6 months were exclusively breastfed, including a mere 7% of children under two years who were fed appropriately [8]. The infant and young child nutrition situation, while improving, was sub-optimal even before this outbreak.

The relationship between infectious disease and nutritional status is complex and bi-directional: the symptoms of infection contribute to reduced food intake and weakened immune responses, which in turn make it more difficult for the body to fight the disease [10]. EVD and nutrition are no different. At an individual level, EVD patients typically present with symptoms indicative of worsened nutritional status, such as diarrhea, abdominal pain, vomiting, and anorexia [11]. When community members, including heads of households and primary caregivers, become infected, the consequences of infection on nutrition extend beyond that individual to the community and household where typical infant and young child feeding (IYCF) practices may be disrupted. This is particularly true during outbreak responses, such as Sierra Leone’s, where quarantines for exposed persons may limit the ability of households to access and utilize food using typical food insecurity coping strategies.

Since this outbreak, scholars have proposed solutions to better prepare for, and respond to infectious disease outbreaks at large [12, 13]. Despite the direct physiological effects of EVD on individual nutritional status, as well as the known complexities surrounding household food and nutrition security during outbreaks [14], few nutrition-related solutions have been discussed. Research conducted in both Liberia and Guinea suggests that there was a complex interplay of bio-social and cultural factors that contributed to nutrition-related impacts during this EVD outbreak [15, 16]. It could be surmised then that similar interrelated processes throughout the food system affected nutrition in Sierra Leone as well.

Improving response and preparedness options warrants an in-depth reflection on lessons learned from this EVD outbreak and response, including a focus on the non-clinical forces that may have influenced nutrition. In Sierra Leone, research has provided evidence for EVD impacts on market chains and trade, yet questions remain about the downstream effects on the nutrition situation during this outbreak and strategies to mitigate it in the future. Considering the entire food value-chain, from food production to retailing and consumption, may offer some answers [17].

Therefore, we firstly sought to explore how and through what pathways the EVD outbreak impacted nutrition in Sierra Leone. Secondly, we investigated the factors to effective implementation of nutrition response strategies during this outbreak. Thirdly, we aimed to use findings to consider a nutrition preparedness and response framework in planning for future outbreaks of this nature.

## Methods

### Research design

This qualitative study collected data over two iterative phases from multiple participant groups using semi-structured, in-depth interviews between September and November 2016. In phase 1, we explored the perceptions of policy makers, hospital management, and responding agency staff employed in Sierra Leone during or since the EVD outbreak. In phase 2, we investigated the community perspectives by interviewing EVD survivors, front-line health workers, and community leaders.

### Sampling

Participants were recruited by a local non-government organization (FOCUS1000) with previous experience conducting qualitative research related to nutrition and the EVD response. Using a criterion-based, purposive sampling strategy, participants were initially identified for interviews choosing specific characteristics that would allow for a range of perspectives [18]. Phase 1 participants were sampled based on their professional role representing government, hospital management, NGO, or United Nations organizations involved in the outbreak response at the national level; Phase 2 participants were sampled by their community role (e.g. EVD survivor, community leader, health worker) as well as their geographic representation across the four provinces of Sierra Leone.

### Data collection

After recruitment, a locally hired data collection team was trained for 40 hours in qualitative research theory and in-depth interviewing skills. Data collectors were university graduates who spoke both English and Krio, as well as Mende or Temne. Training also included field tests and revisions of the interview guides to ensure appropriate content considering multiple languages. The most qualified interviewers were then chosen for fieldwork, during which FOCUS 1000 senior staff supervised the data collection team and oversaw all study procedures.

Prior to data collection, FOCUS1000 recruited prospective study participants through phone calls, emails, and letters, as well as sought permissions from local authorities for conducting this work. No notable recruitment challenges were reported. Then, over three months, the team conducted interviews lasting between 45 and 60 minutes, primarily in English and Krio language, and to a lesser extent in Mende and Temne. Two semi-structured guides were used to guide the interviews in subsequent phases, each with similar overall content yet different question types and probes (Table 1).

**Table 1:** Semi-structured interview guide content by study phase and type of participant.

	Phase 1	Phase 2
	*Key Informant* Guide Content	*Community Informant* Guide Content
**1.**	EVD outbreak impact (general)	EVD outbreak impact (general)
**2.**	EVD outbreak impact (on organization)	Perceptions of nutrition care to EVD patients
**3.**	EVD outbreak impact (on health system)	Perceptions of nutrition support to EVD survivors
**4.**	EVD outbreak impact (on nutrition services within stakeholder organization)	EVD (outbreak, disease, containment) impact on infant and young child feeding
**5.**	Quality of support for nutritional health	Recommendations and lessons learned
**6.**	Nutritional support using interim guidelines^a^	
**7.**	Coordination and information sharing	
**8.**	Recommendations and lessons learned	

^a^Interim guidelines provided to countries by the global health organizations to address nutrition care and treatment in the context of EVD [19].

The primary content domains within the interview guides were chosen *a priori*, based on a review of the literature, the guiding research questions, and in agreement with UNICEF and FOCUS 1000 team members who had experienced the outbreak. Within those broad domains, though, specific questions and probes, particularly those of the phase 2 informant guide, were derived from phase 1 data in an iterative and flexible research design that allowed for triangulation between phases and participant types.

During **Phase 1**, 21 interviews were conducted among *key informants*, government policy makers, as well as hospital management and programme agency staff of UN agencies and non-government organizations, who could speak not only to their own personal experiences but also to those of the organizations they represent. In **Phase 2**, 21 additional interviews were conducted among *community informants*, including EVD survivors, front-line health workers, and community leaders, using a second interview guide which focused on understanding individual lived experiences.

Interviews in each phase were conducted with the goal of reaching data saturation, until a repetition of key themes, at which point no additional data collection was thought to yield new information relevant to the research questions [20]. All but one interview was digitally recorded. Each transcript included field notes written by a data collector immediately after interviewing [21].

### Data analysis

All digital recordings and corresponding field notes were translated and transcribed into English. Transcripts were continually reviewed and spot checked for accuracy by members of the FOCUS1000 team. Inconsistencies between recordings and transcriptions were resolved by the team prior to analysis.

English transcripts were then uploaded into Dedoose qualitative software for data management and analysis [22]. A codebook was also developed based on both the initial study objectives and interview guide content. It served as the analytic framework within Dedoose and contained 46 codes across 7 thematic areas.

The coding procedures followed a systematic process in a team-based approach using 3 data analysts [23]. After codebook development, we conducted inter-coder reliability testing within Dedoose to ensure consistency across individual coding efforts [24]. Pooled kappa scores of 0.76 and 0.71 during reliability testing ensured that we had ‘good’ internal consistency prior to coding the entire data set [25, 26]. The analytic team also held weekly meetings to address any challenges and questions to ensure continual alignment throughout the coding process.

Using 46 codes and sub-codes across 42 transcripts, a combined 1918 code applications were made by the analytic team across the entire data set. Both data-driven and theory-driven codes were identified during this process [27]. Those code applications, which labelled thematic units of text, were then stratified and extracted using Dedoose [22]. Salient themes and sub-themes were identified during interpretation and presented as a combination of tables, figures, and quotations to best illustrate findings [28]. They were then presented to the data collection team, as well as to representatives from government, civil society, and non-government organizations, in a participatory workshop format. This process of ‘member checking’ provided a forum for feedback and discussion around the findings, as well as confirmation that our interpretations of the data accurately represented the nutrition impacts felt during the outbreak [29].

### Ethical approval

The study protocol was approved by the Office of the Sierra Leone Ethics and Scientific Review Committee. All participants provided oral informed consent prior to interviewing and no identifiers were collected as part of study procedures.

## Results

Overall, 42 individuals participated in this study, including 21 *key informants* and 21 *community informants* (Table 2). The 21 *key informants* were recruited from various organizations, including 9 government or policy-level representatives, 6 non-governmental organization staff, 4 United Nations staff, and 2 hospital managers. The 21 *community informants* included 10 EVD survivors, 9 health workers, and 2 community leaders. All 4 of Sierra Leone’s regions were represented approximately equally. The sample was approximately 60% female, with a lower proportion of female *key informants* than *community informants*.

**Table 2:** Socio-demographic characteristics of study sample.

All participant characteristics	
No. of participants, n	42
Female, n (%)	25 (59.5%)
***Key Informant* characteristics**	
No. of key informants, *n* (%)	21, (50.0%)
Female, *n* (%)	7 (33.3%)
Type of organization, *n* (%)	
Government	9 (42.9%)
United Nations	4 (19.0%)
Non-government organizations	6 (28.6%)
Hospital Management	2 (9.5%)
Years spent in professional role, median (min, max)	6.5 (2–27)
***Community Informant* characteristics**	
No of informants, n (%)	21 (50.0%)
Female, *n* (%)	18 (85.7%)
Type of informant, *n* (%)	
Survivor of Ebola Virus Disease	10 (47.6%)
*Primary caregiver*	5 (23.8%)
*Other household member*	3 (14.3%)
*Nurse or nurse aide*	2 (9.5%)
Health worker (medical doctor, nurse, front-line health worker, midwife)	9 (42.9%)
Community leader	2 (9.5%)
**Geographic regions and districts, *n* (%)**	
Southern Province (Bo)	10 (23.8%)
Eastern Province (Kenema)	11 (26.2%)
Northern Province (Port Loko)	12 (28.6%)
Western Area (Freetown)	9 (21.4%)

Overall, all participants explained that Sierra Leone felt widespread and profound impacts from EVD across sectors.

“*What is clear*, *like I mentioned*, *work came to a halt in this country…there was nothing happening*, *and then (that) means everything was affected*. *There was no schooling…there was no agriculture farming…health services were very bad… everything came to a halt*. *So where things came to a halt in a country*, *for nearly two years…then anybody can imagine the impact of that kind of problem*.”

-High-level government official, *key informant* interview

### Nutrition-related impacts

**Fig 1:**
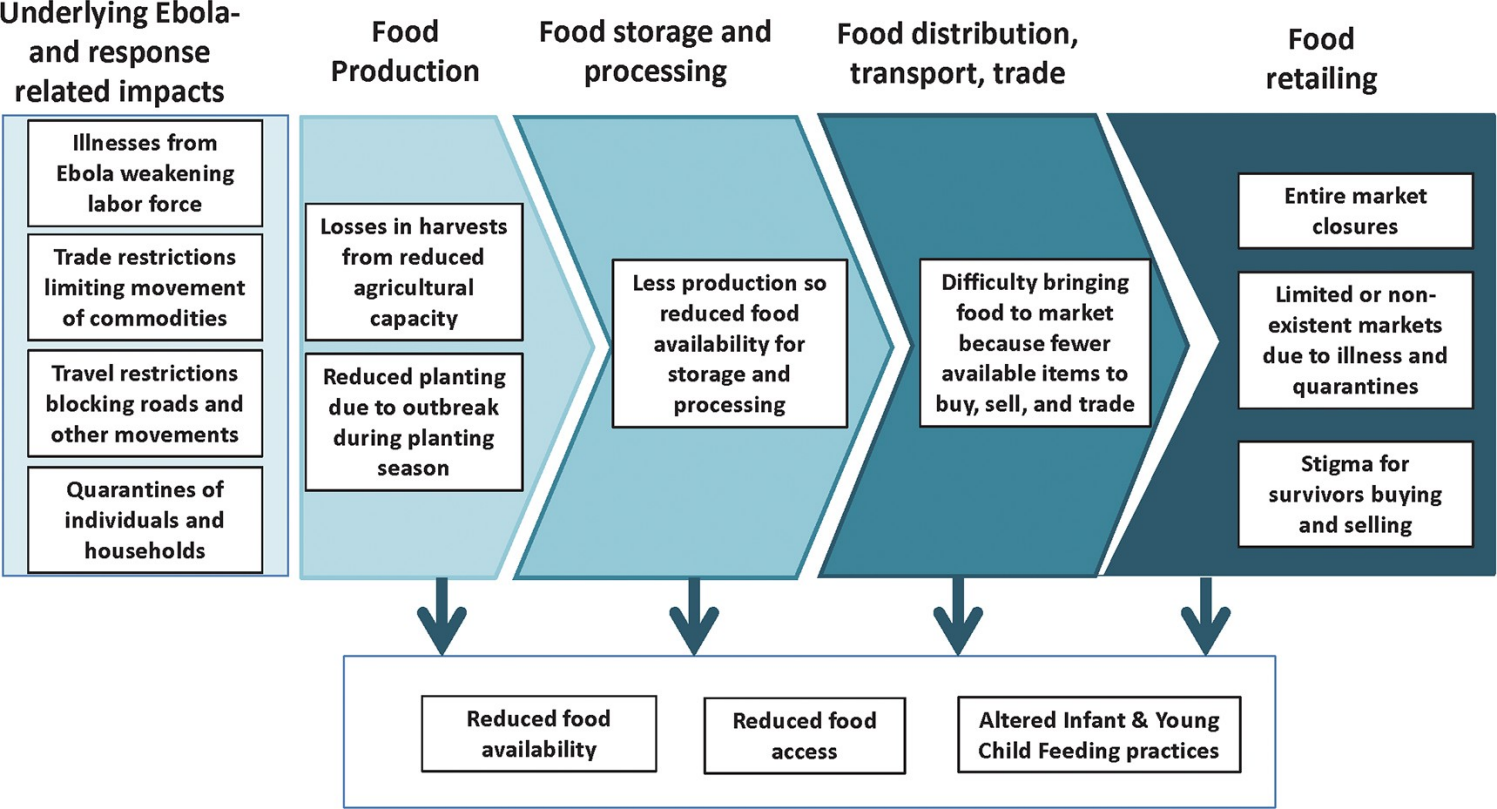
Emergent themes highlighting key EVD impacts along abbreviated food value-chain.

Data suggest that EVD impacted the entire food value-chain, ultimately affecting individual-, household-, and population-level nutritional status. These impacts were multi-factorial: from the clinical manifestation of the disease itself, from the outbreak consequences, and from the containment measures/response strategies. To reduce the spread of EVD in Sierra Leone, the government restricted people's movements by blocking roads and imposing household and community quarantines. However, in doing so, food security and nutrition were negatively impacted due to upstream market chain disruptions (Fig 1).

*“…socio-economically the impact of the EVD outbreak was and is still really great; because let’s say for example you have a whole value chain*, … [and as] *there wasn’t much production going on during the EVD era*, *people were not able to go to their farms*, *people were not able to harvest their farms*, *people were not able to process*, *people were not able to go to the market you know*, *and when you talk about nutrition you need to look at the quality and quantity of food intake*, *you have to look at the number of times and the timeliness of the food intake…”*

-Female program manager, *key informant* interview

“*Most of them were not able to survive because of lack of food availability in the community due to the lockdown…and there was no movement of people from district to district. Therefore, food was scarce and hard to get. Also, money was the other challenge…to buy baby food in the shops…for those who could afford [to buy it]…most work places were closed. And most mothers and family heads were not working*.”

-Female front-line worker, *community informant* interview

Fig 1 forms the basis of the subsequent sub sections, which are organized below by emergent themes identified across the food value-chain from *Food Production* to *Food Retailing*.

#### Food production

A primary nutrition-related impact was at the food production level of the food value-chain, as restriction of movement, reduced workforce mobility, and migration of people to safe zones all impacted domestic agriculture. Participants explained that there were great losses in harvests from reduced manpower stemming from the outbreak, which coincided initially with the planting season. Individuals who once helped in agricultural activities were oftentimes too sick or quarantined and thus unable to assist in cultivation, including planting and harvesting activities. *“I was the bread winner so when I got EVD*, *living became difficult for my entire family*.*”* explained one EVD survivor. The quarantine was a particularly formidable challenge for rural community members because agricultural activities were described as largely communal in nature.

#### Food storage and processing

Data suggested that with reduced food production came less food availability for storage and processing. Especially in households that were quarantined, participants explained that stored foods needed to last for longer periods than usual since food sharing and barter was not possible during the outbreak.

“*We were restricted, especially when we were quarantined…when they would quarantine people they would give us half a bag of rice… …if your house is lucky to go through the 21 days (EVD) free then the security will now allow you to go get food. If not, we would be (quarantined) for weeks or months…. It was really not easy for us then…*”

-Female EVD survivor, *community informant* interview

These response measures were reportedly a foremost challenge due to community reliance on inter-household food sharing as a primary coping strategy in times of food shortages.

#### Food distribution, transport, and trade

Participants explained that during the outbreak bringing food products to market was difficult because there were fewer surplus food items available to move, sell, buy, and trade as a result of reduced agricultural production. Coupled with a 20% reduction in the number of working traders during the outbreak, both travel and trade restrictions on imports and exports further reduced commodity flows (e.g. exporting parboiled rice to Guinea was difficult). These effects were described by participants in this study, as well as in FAO-supported research detailing the regional effects of malfunctioning market chains and haphazard flows of agricultural products during this outbreak [7].

#### Food retailing

Finally, fewer people could travel to markets due to illness and restrictions. There were also entire market closures (e.g., Luma market), including community auctions, during the outbreak. Even among EVD survivors during and after the outbreak, participants explained that stigma was a barrier to both buying and selling food products.

*“…People fear us because we survived EVD so if they interact with us they* [think they] *could contract the virus*. *…if we went to the market to buy some food*, *they would not sell to us because we are survivors*.*”*

- Female EVD survivor, *community informant* interview

Consequently, the entire food value-chain was disrupted resulting in negative impacts on various dimensions of food availability, food access, and food utilization during the outbreak.

### Largest nutrition-related challenge for responding organizations

The most prominent nutrition-related challenges described by *key informants* could be organized into three primary categories: a) food availability and access, b) capacity, coordination, and logistics, and c) screening malnutrition cases (Table 3).

**Table 3:** Summary of nutrition-related challenges reported by organizations.

Nutrition-related challenges reported by organizations	Specific reasons for and examples of primary challenges
Primary themes	Secondary themes
**Lack of food availability and reduced food access**	• Market disruptions from reduced food production and travel restrictions
	• High demand & low supply of foods
	• Higher food prices
**Inadequate capacity, coordination, & logistics**	• Low prioritization of nutrition within organizations–money diverted to response activities
	• Difficulties delivering food assistance to hard-to-reach, rural communities
	• Safety concerns during food delivery to communities
**Reduced screening for malnutrition cases**	• Travel restrictions limiting people’s movement
	• Reduced health-seeking behaviors from less movement and distrust
	• Early denial of outbreak and distrust among community

#### Food availability and access

First, key informants explained that food availability was a serious challenge for the entire community due to a combination of reduced food production, higher demand, and higher prices.

*“During the outbreak*, *everything including food*, *became expensive and hard to get*. *The little food* [available] *in the market was in high demand…and at the height of the EVD outbreak*, *people were afraid of one another’s body contact*, *as the slogan says ‘‘don’t touch” was in the mind of everyone…As everyone was afraid of the virus in a gathering because people were of the view that if one goes out*, *the risk of contracting the virus is very high*, *especially when stakeholders were warning people to stay home*. *Food was short* [supply]*…and it was very expensive*. *For instance*, *foods items that were sold and bought for SLL 1000* [0.18 USD] *were sold for SLL 2000* [0.36 USD].*”*

Female nutritionist, *key informant* interview

Second, while food rations were distributed by humanitarian organizations, there were reports of either expired rations or inadequate supply of rations in specific localities. Participants explained that there were inadequate supplies because the rations were initially only intended for EVD-affected individuals, but due to food production losses, entire communities were suffering from heightened levels of food insecurity.

*“One of the biggest challenges that we had to deal with was the fact that when we paid visits to a particular community with the intention to only feed an exact* [specified] *number of beneficiaries on a list*, *but having (in reality) to provide the food throughout the (whole) community because everybody needed it*. *That was the most frustrating challenge we had and it kept continuing … you will meet another 50 more vulnerable children waiting around the same center for the same food…”*

-Male nutrition manager, *key informant* interview

#### Capacity, coordination, and logistics

Second, key informants identified the low prioritization of nutrition activities within their organizations as a barrier. They explained that early during the outbreak, nutrition activities were oftentimes removed from entire work plans to focus the response solely on EVD containment and treatment activities.

“*I will say that we completely shifted our operations from nutrition activities that we were doing to focus mainly on EVD*.”

-Male stakeholder, *key informant* interview

For those nutrition activities and food distributions that were being implemented, safe and adequate transport at the community level, especially in hard-to-reach, rural areas, remained a continual challenge Some participants also indicated that food deliveries were subject to both harassment and attacks by community members at times of heightened food insecurity and when there was confusion as to why some individuals received food assistance but others did not.

#### Screening malnutrition cases

In addition, albeit to a lesser extent, participants explained that there were increased difficulties screening child malnutrition cases due to travel restrictions, reduced health-seeking behaviors, and early denial of the outbreak by local community members.

### Coping strategies for nutrition challenges

Both internal and external inputs were identified to be important coping strategies in the face of those primary nutrition challenges.

#### Better planning and coordination efforts

The most salient theme that emerged from the interview data was that the planning and coordination of the EVD response improved from the beginning until the end of the outbreak. Key informants ascribed improvements in the nutrition response to developing district-level working committees, planning carefully for food distributions (e.g., military protection, reservations of vehicles, inter-stakeholder coordination), and coordinating technical nutrition actions during the outbreak through the Nutrition Emergency Multi-sectoral Taskforce, of which the Scaling Up Nutrition (SUN) secretariat was one coordinating body involved to maximize program coverage, impact, and to reduce duplication. This was important because after the outbreak began, participants explained that the surge of new international organizations arriving to Sierra Leone, where existing bodies had already been working on health and nutrition issues using their own coordination mechanisms and defined roles, contributed another layer of planning and coordination complexity. Community members also indicated that their arrival contributed to the overall climate of confusion because it was unclear what unique services were available by organization.

#### Enhanced social mobilization and engagement activities

At the community level, meetings between the humanitarian/medical community and local community members were thought to be essential for ensuring smooth implementation of any nutrition activities, including more timely and safer food distributions.

“*…each time we faced difficulties, we called on the community stakeholders (leaders) and asked them to talk to their people to reason with us by telling them (community members) who the food was specifically meant for…*”

-Male nutrition manager, *key informant* interview

This collaboration between the humanitarian/medical and local communities was important for improving relations and ensuring nutrition activities were delivered more successfully.

#### Provision of nutrition technical capacity and food assistance

Finally, participants identified the provision of additional nutrition capacity and increased food assistance over time as external inputs enabling the community to overcome nutrition-related challenges. The great influx of donor resources soon following the August, 2014 declaration of the WHO that the outbreak was an *international public health emergency* contributed to the requisite nutrition capacity and availability of direct food assistance needed in Sierra Leone for an improved nutrition response [30].

#### IYCF impacts

IYCF practices were negatively influenced as children were separated from infected mothers. Also, sick mothers and EVD survivors were instructed to stop breastfeeding in the beginning of the outbreak since there were no clear ‘IYCF during EVD’ guidelines that had been developed at that time. As such, front-line workers and caregivers faced challenges modifying breastfeeding, complementary feeding, and caregiving practices.

#### Breastfeeding practices

Participants explained that breastfeeding fully stopped in many cases because of ‘no contact’ instructions for infected caregivers and their young children. At treatment centers, sick mothers were separated from their infants “*if they feel sick*” and thus were instructed to no longer breastfeed. Key informants expressed the challenge behind this policy, though, in the wake of the outbreak.

“*…there was a no-touch policy…then the question arose, if your child is breastfeeding and we are supposed to promote exclusive breastfeeding, I mean tell me how…what are we going to do?*”

Male high-level stakeholder, *key informant* interview

Community informants explained that breastfeeding practices largely persisted without interruption early on during the outbreak.

“*There was a lady who was breastfeeding while she contracted the disease. She continued giving the breastmilk to the child until the child also became infected. Both the mother and child died …*”

-Female front-line worker, *community informant* interview

After more coordinated and enhanced sensitization efforts were carried out, it became more socially acceptable to stop breastfeeding if the mother or child were infected.

#### Complementary feeding and food substitution

Due to heightened economic challenges and resulting household food insecurity, participants explained that complementary feeding practices changed in important ways during the outbreak. The two most frequently-mentioned coping strategies to feed young children included food substitutions with more affordable, yet less nutritious options and food quantity reduction due to lack of food access.

“*There was limited food for the children to eat. If the parent was used to feeding the children with three meals a day, during Ebola there was not enough food so it was limited to one or two meals a day*.”

-Female health worker, *community informant* interview

Ready-to-use-therapeutic foods for treatment of acute malnutrition, as well as other food items including milk substitutes, corn soy blend, and entire food baskets were also provided at no cost from humanitarian organizations, which were, “*concerned with their* [community] *welfare*”.

#### New caregivers and feeding styles

During the outbreak, there was an increase in the number of orphaned children and children whose primary caregiver was absent for extended periods for treatment or permanently because of death. This gap in caregiving necessitated that extended family members (e.g. grandmothers, relatives, and in-laws) feed and care for young children that were not their own, resulting in multiple challenges.

“*When I got Ebola and was taken to the treatment center, it was only my mother who was serving as a caregiver. I left some market [items] at home…it was from those sales that my mother was using to buy food for my child for feeding … also for feeding of my family. I was the breadwinner so when I got Ebola living became difficult for my entire family*.”

-Female EVD survivor, *community informant* interview

In the post-EVD context, a front-line worker explained that he has seen more positive IYCF practices resulting from the sensitization and messaging efforts during the outbreak, specifically in relation to hand washing practices and breastfeeding.

### Response acceptability and suggestions for improvement

Data suggest that the level of community acceptability toward the interventions during the nutrition response improved over time as trust was built through enhanced sensitizations and more appropriate social and behavior change communications (SBCC). Specifically, themes around therapeutic foods, including infant formulas, interim care guidelines, and SBCC emerged in this area of inquiry.

#### Food assistance

The food assistance provided by the humanitarian community took many forms, including the provision of ready-to-use foods (e.g. PlumpyNut^®^) and infant formulas (e.g. F-100) for addressing acute malnutrition, as well as food baskets with local foods such as rice, and other specialized nutritious foods (e.g. SuperCereal Plus) for general household nutrition security [31].The World Food Programme (WFP) was reaching people at Community Care Centers with food baskets including rice, SuperCereal Plus, pulses, salt, and vegetable oil intended to feed families of 5 persons/month [32]. Overwhelmingly, interview data indicate that the food assistance was highly accepted and much needed. Both infant formulas and specialized nutritious foods were perceived to be “*safe*”, “*nutritious*”, “*important for addressing malnutrition*”, “*improve growth*”, and “*overcome illness*” among others. Front-line workers indicated that some children who were used to breastfeeding took some time to adjust to using the ready-to-use infant formulas but mostly did so without too much trouble.

*Community informants* explained that the sharing and selling of food assistance was not uncommon.

*“Some mothers were even using the opportunity to frequently go to the hospital to just have more supply and at the same time keep them* [food products] *at home for domestic use or for sale…because some people were deliberately selling the biscuits to diamond miners and other people in villages*, *and used the money to buy other food items…”*

-Male community leader, *community informant* interview

It is unclear to what extent sharing of specialized nutritious food occurred, but one front-line worker explained that in her opinion, “*Honestly*, *I will rate it 40%* [appropriate usage] *because 60% of all ready-to-use foods are in the market for sale*.”

#### Interim nutritional treatment and care guidelines

Most stakeholders interviewed were not involved directly with nutritional treatment and care. However, interview data among key informants suggest that interim guidelines usage was mixed. While the guidelines were reportedly useful for nutritionists and clinical staff on the front lines, not all management staff were aware of them. Some *key informants* suggested that both their own organizational nutrition guidelines and these interim guidelines were being used concurrently. Just as many key informants indicated positive perceptions toward the guidelines, though.

#### Social and behavioral change communications

The nutrition-related SBCC efforts were said to be largely positive because they were multi-channeled, including numerous forms of print, interpersonal, and mass media, as well as were culturally appropriate and understandable.

“*That is why most of the programs were done in our local languages, and backed up with messages with pictures that showed what to do and what not to do. If it was not for those messages that were very frequent, we would have lost lots of children…but it really helped…and I like the way that everybody accepted the messages*.”

-Female EVD survivor, *community informant* interview

The combination of varied SBCC channels was appreciated because it allowed for messages to have a wide reach. Some individuals indicated not listening to radio but receiving information at health clinics; others explained that radio was effective for their communities. Some themes emerged in relation to the persistent lack of food access despite well-received IYCF messaging. Importantly, participants also explained that the SBCC approaches were acceptable and important, but not timely–coming late in the response as an afterthought or as a complementary, rather than as a primary, response approach.

In terms of on-going behavior change still today, participants had mixed perceptions with some individuals indicating that behaviors “*went back to normal*” after Sierra Leone was declared EVD free and others indicating that exclusive breastfeeding and water, sanitation, and hygiene behaviors persist today.

### Lessons learned from different perspectives

Finally, comparing the community leaders, survivors, caregivers, and frontline workers who represented the voice of the community members to the Government, United Nations, and NGO staff who provided policy and organizational voices, lessons learned underscore unique perspectives for consideration (Table 4).

**Table 4:** Comparison of differential perspectives on the same outbreak response.

	Exemplar quotes comparing unique perspectives toward important lessons learned	
**Community members, health workers**	“*Even though I am not wishing any outbreak for our nation in future*, *but we plead to the government and international community to learn how to respond quickly to any form of disease that they hear about…And the response should be at once*, *because any attempt to delay will cause some problems and it may exceed the expected limit*. *Let the health system be really strengthened for any form of illness*.*”* -Female front-line worker, *community informant* interview “*Working together was a very big boost in fighting Ebola in a country where everybody came with one focus to defeat Ebola and it worked well*. *Engaging the people in ensuring that they participate in their own programs was also a very good thing… everybody was involved…that was why we were able to end Ebola in the country*. *Using local knowledge is very*, *very important you know…these were some of the lessons learned from the Ebola outbreak*.*”* -Male high-level stakeholder, *key informant* interview	**Government, United Nations, NGO staff**

*Key informants* discussed that a key learning (other than coordination) was the true importance of community involvement for an effective response. This was not a key lesson learned for *community informants* who were already aware of their own importance; for them, the key lessons learned focused on timely government support, adequate health worker capacity, and the importance of having a strong health and food system better equipped to absorb such events in the future.

## Discussion

Through in-depth interviews with individuals who directly and indirectly lived through the EVD outbreak in Sierra Leone, we gain a more refined understanding of the potential pathways through which the food system and infant and young child nutrition was impacted. Participants in this study provided multiple perspectives that described humanitarian response strategies which, coupled with the outbreak, disrupted livelihoods limiting food production and trade, weakened typical coping mechanisms, and altered care practices for infants and young children, including orphans.

Our qualitative data revealed that the EVD outbreak impacted food security and nutritional status through each level of the food value-chain [33]. The scope of this impact was unique, though, compared to that of other infectious disease outbreaks: it disrupted entire health and food systems. In that sense, its systemic effects were more similar to the 2010 earthquake in Haiti than to the resulting cholera outbreak that same year [34]. Cholera did not have the system-level disruption that EVD did across Sierra Leone, Liberia, and Guinea, despite high morbidity and mortality [35]. In that sense, the EVD outbreak more closely resembled a disaster, such as the earthquakes in Nepal (2015) and Haiti (2010) where access to basic needs, including food and nutrition, were a foremost priority [36, 34]. Outbreaks on par with EVD, while rare, may conceptually draw from disaster responses where many lessons have already been documented, including the need for agile and adaptive systems, enhanced technologies, community engagement, local ownership, and strong coordination mechanisms are paramount [37, 38, 39].

To ensure a resilient food value-chain, enhanced safety-net approaches at each stage are critical for ensuring actors can continue pro-nutrition activities at each stage, even during disruptions of this magnitude. Our findings highlight this need not only on the agriculture, food production side of the chain but also downstream at household and individual levels where business incomes and resultant household food expenditures were profoundly hurt [40]. In support of other published work from this event, our findings underscore the importance of trusted community-level nutrition expertise, ideally complemented by capacity in the social and behavioral sciences and community engagement, for improved responses [41, 42]. Relevant lessons learned can be drawn from other infectious disease outbreaks, such as the HIV/AIDS epidemic that has left a generation of orphaned children facing similar health and social challenges to those felt in the wake of this EVD outbreak [43]. Well-coordinated, interdisciplinary approaches have potential to marry the typically distinct concepts of clinical care and treatment to culturally-appropriate social and behavioral intervention strategies for more appropriate and acceptable implementation both during and after such emergencies. The strong community distrust and social resistance to public health intervention during this response underscore the complexity of such outbreaks and the need for respectful and well-planned response measures [44, 45].

Regardless of planning, there will always be consequences to response measures during this type of complex emergency. Our findings highlighted some of these unintentional effects, for instance how typical household food insecurity coping strategies during market fluctuations or poor harvests were made difficult by the imposed 21-day quarantines. The quarantines placed additional economic and social burdens that contributed to individual and household diet-related challenges, above those felt by the disease; such lessons should be used to reflect on the viability of alternative approaches, such as Community Care Centers [46]. This finding is particularly important for nutrition in interdependent cultural contexts where food sharing, bartering, and communal agricultural activities are the keystones of rural livelihoods [47]. Our findings suggest that the ‘dynamic complexity’ of food and nutrition insecurity becomes more complex during periods of outbreak [14], thus requiring careful, proactive preparedness across the value-chain. Such planning among both the organizations already present in a country and those newly arriving in the wake of such a disaster response will present serious coordination challenges that need to account for these new actors, as well as increased sensitization to clearly inform community members what organizations are providing what specific services. Regardless, our findings illustrate why preparations should be made across the food system, including the development of agreed-upon standard operating procedures to guide coordinated food and nutrition activities when movement restrictions are put in place during outbreaks, from production (e.g., provision of seeds to households to help during planting when trade is not possible) through retailing (e.g., creation of temporary markets deemed ‘safe’ with provision of food assistance).

Finally, despite improved coordination efforts, enhanced food assistance, and a more aligned humanitarian response over the course of this outbreak, our data suggest that nutritional challenges were disproportionately felt by infants and young children–an already nutritionally-vulnerable group. Breastfeeding, complementary feeding, and caregiving practices were all impacted in Sierra Leone, both directly by EVD and indirectly through market disruption and due in part to response measures put in place for disease containment. Balancing future responses to both curb contagion while also reducing the potential for negative nutritional consequences likely will remain a challenge considering the close relationship between the two. Utilizing standardized nutrition guidelines, like those provided for treatment and care during this outbreak, may prove to be a useful starting point that can assist front-line workers [48]. Also, by focusing response efforts higher up the food value-chain through both nutrition-sensitive approaches and the implementation of social safety nets, a future response may be able to better avoid some of the IYCF challenges reported in our study. Thus, agricultural activities and nutrition-sensitive intervention approaches that sustain adequate food production [49], could be considered preparedness strategies.

This qualitative study had several important strengths. First, it included both participant and analytic triangulation, a key study aspect designed to improve the data credibility [50]. Second, data collection was iteratively carried out over two phases. This two-phase design allowed for an extended period of data collection where interviewers conducted semi-structured interviews based in part on findings from one participant to the next. Third, this study uniquely considered the perspectives of the interviewers through textual analysis of their detailed field notes following interviews, which is a core aspect of ethnographic research [51]. Fourth, by using ‘member checking’, whereby we presented final study results to key stakeholders and data collectors in a participatory workshop, we could be more confident that our interpretations of the textual data accurately reflected the accounts within them [29].

However, this research also included some limitations. Many key informants who had worked in Sierra Leone during the EVD outbreak were no longer in those same positions and could not be interviewed. However, in those cases, we recruited participants who were either direct successors to those staff or other key informants who were in similar positions during the outbreak. Similarly, we interviewed participants in 2016 about events from previous years; memories and perspectives of past events can change over time, a primary reason we corroborated findings with multiple participants and using secondary data sources. Further, those people who were interviewed survived the outbreak. While we heard both positive and negative accounts of individual experiences, it is possible that those individuals who did not survive EVD may have had different perspectives. By interviewing family members of those deceased, as well as EVD survivors, we believe that we provided a comprehensive account from multiple angles nonetheless.

## Conclusions

This study underscores the magnitude of this EVD outbreak, which considerably impacted Sierra Leone’s food value-chain, negatively affecting food availability and food access across regions. At the policy level, adequate investments in improved emergency outbreak preparedness across the food value-chain may put nutritional health systems in more timely and coordinated positions to address not only the direct threat of an infectious disease but also the indirect toll that poor nutrition takes on community health. At the organizational level, nutrition programme planners should ensure necessary resources and capacity to ensure a combination of nutrition-specific and nutrition-sensitive response options prioritizing community involvement in design, coordination, and implementation across the food value-chain. These important lessons learned should be adapted to other contexts, such as the Democratic Republic of Congo, where a very similar EVD outbreak has been occurring since 2018 [52]. Disasters of this magnitude may unavoidably face food security challenges, yet taking an interdisciplinary approach by convening experts from disciplines across the value chain for preparedness planning, for instance, may help to mitigate such widespread nutrition effects in similar complex emergencies.
